# Training and validation of a novel non-invasive imaging system for ruling out malignancy in canine subcutaneous and cutaneous masses using machine learning in 664 masses

**DOI:** 10.3389/fvets.2023.1164438

**Published:** 2023-09-29

**Authors:** Gillian Dank, Tali Buber, Anna Rice, Noa Kraicer, Erez Hanael, Tamir Shasha, Gal Aviram, Amir Yehudayoff, Michael S. Kent

**Affiliations:** ^1^HT BioImaging Ltd., Hod Hasharon, Israel; ^2^Department Biomedical Engineering, Tel Aviv University, Tel Aviv-Yafo, Israel; ^3^Department of Mathematics, Technion, Haifa, Israel; ^4^Department of Surgical and Radiological Sciences, School of Veterinary Medicine, University of California, Davis, Davis, CA, United States

**Keywords:** cancer, diagnostic, artificial intelligence, neoplasia, machine learning

## Abstract

**Objective:**

To train and validate the use of a novel artificial intelligence-based thermal imaging system as a screening tool to rule out malignancy in cutaneous and subcutaneous masses in dogs.

**Animals:**

Training study: 147 client-owned dogs with 233 masses. Validation Study: 299 client-owned dogs with 525 masses. Cytology was non-diagnostic in 94 masses, resulting in 431 masses from 248 dogs with diagnostic samples.

**Procedures:**

The prospective studies were conducted between June 2020 and July 2022. During the scan, each mass and its adjacent healthy tissue was heated by a high-power Light-Emitting Diode. The tissue temperature was recorded by the device and consequently analyzed using a supervised machine learning algorithm to determine whether the mass required further investigation. The first study was performed to collect data to train the algorithm. The second study validated the algorithm, as the real-time device predictions were compared to the cytology and/or biopsy results.

**Results:**

The results for the validation study were that the device correctly classified 45 out of 53 malignant masses and 253 out of 378 benign masses (sensitivity = 85% and specificity = 67%). The negative predictive value of the system (i.e., percent of benign masses identified as benign) was 97%.

**Clinical relevance:**

The results demonstrate that this novel system could be used as a decision-support tool at the point of care, enabling clinicians to differentiate between benign lesions and those requiring further diagnostics.

## Introduction

Cancer is the leading cause of death in 45–47% of dogs over the age of 10 (1, 2). Dobson et al. reported on the incidence of canine tumors in the UK. The skin and soft tissues were the most common sites for tumor development, with a standardized incidence rate of 1,437 per 100,000 dogs per year (3). Merlo et al. reported that mammary cancer was the most frequently diagnosed cancer in female dogs in the cancer registry in Genoa, Italy, accounting for 70% of all cancer cases. The reported incidence of all cancers was 99.3 per 100,000 dog-years in male dogs and 272.1 in female dogs (4). Baioni et al. reported on the cancer incidence in the Piedmont Canine Cancer Registry. They collected data on 1,175 tumors confirmed by histopathological diagnosis. The incidence rate was 804 per 100,000 dog-years for malignant tumors and 897 per 100,000 dog-years for benign tumors (5). Graf et al. reported on the incidence rate in the Swiss canine cancer registry. The most common tumor types were mast cell tumors, lipomas, hair follicle tumors, histiocytomas, soft tissue sarcomas, and melanocytic tumors, with >1000 tumors per tumor type. The average incidence rate of all tumor types across the 227 registered breeds was 372.2 (6). Martins et al. performed a 7-year retrospective study on 1,185 cases diagnosed as cutaneous tumors, with 62.9% classified as benign and 37.1% as malignant (7).

Dogs with undiagnosed malignant neoplasia are routinely seen in general veterinary practice settings. There is a great need to improve our diagnostic capabilities, thus improving the ability to diagnose cancer and provide better treatment (2). Fine-needle aspiration or biopsies are the recommended diagnostic tests for subcutaneous and cutaneous masses (8). While histopathology is the gold standard for diagnosis, in many cases, clinicians prefer to perform fine-needle aspirates for superficial masses because they are less invasive and less expensive. Despite the ease of fine needle aspiration, studies have shown only an 80% retrieval rate on cytology (9, 10). Therefore, an additional non-invasive, simple-to-use procedure for early cancer detection would benefit veterinarians, clients, and dogs (11).

Thermography is a process by which a thermal camera captures and creates an image/video using infrared radiation emitted from the tissue. Tissues have different thermophysical and heat transfer rate properties that are affected by the compositions, morphology, density, heat capacity, and vascular networks (12–16). In veterinary medicine, the only studies performed evaluated steady-state thermography in tumors (17–20). Mast cell tumors were shown to be colder or warmer than non-tumoral areas (17). Mammary tumors and appendicular osteosarcoma showed elevated tumor temperatures relative to normal tissue (18, 19). Another study evaluated the temperature differences in benign and malignant circumanal gland tumors and found that tumors were colder than healthy sphincter skin (20). This supports the premise that cancer cells have different thermal properties compared to normal tissues; however, the results differed based on the tumor type (17, 19). The three studies concluded that while infrared thermal imaging cannot be used as the sole diagnostic tool, it may be a good ancillary diagnostic modality and that further investigation would be necessary to determine the impact of this technique when adopted clinically (18–20). Thermography has also been evaluated in human tumors, including breast cancer, without sufficient evidence that it can be used as a screening tool (21). Steady-state thermography has shown mixed results in differentiating between malignant and benign tissues.

AI has emerged as a transformative technology in thermography, offering considerable advancements in tumor detection. AI-based algorithms have significantly improved the accuracy and sensitivity of thermal anomaly detection, enabling early identification of potential tumor regions (22). AI-driven thermography techniques have also demonstrated remarkable success on large-scale datasets in differentiating between benign and malignant tumors, thereby aiding in the characterization and classification of tumors (23).

The HT Vista system is composed of a control unit which includes a mini personal computer, a touch screen, a dedicated software application, and a handheld probe. The probe consists of an optical camera, a high-power LED (Light-Emitting Diode) emitter (i.e., the heating source), and an inherent LWIR (long-wave-infra-red) thermal video camera, which records the temperature throughout the scan. Unlike other methods mentioned above, which measure only the tumor baseline temperature and require a thermally controlled environment (e.g., stable room temperature) or are affected by the presence of windows or outside temperature, HT Vista measures the difference in the temperatures between the mass and the adjacent normal tissue at baseline and throughout the scanning process (the heating and cooling phase) and is, therefore, less affected by the external environment. The data are sent to the service cloud, where they are classified using a machine learning algorithm to determine whether the mass requires further investigation (11).

A previous pilot study was performed and showed encouraging results, with an accuracy, sensitivity, specificity, positive predictive value, and negative predictive value of the system of 90%, 93%, 88%, 83%, and 95%, respectively, for all masses in 45 dogs with 69 masses (11).

This prospective validation study aimed to assess the performance of the HT Vista machine learning algorithm in classifying lesions as either benign or as masses that require additional diagnostics.

## Materials and methods

This prospective study was approved by the Israeli Health Ministry ethical review board committee (PET 2023-03-NE).

### The device

The HT Vista system is composed of a control unit that includes a mini personal computer with internet capabilities, a touch screen, a dedicated software application, and a handheld probe. The probe consists of an optical camera, a high-power LED (Light-Emitting Diode) emitter (i.e., the heating source), and an inherent LWIR (long-wave-infra-red) thermal video camera (resolution 19,200 pixels, sensitivity <50 mK, FLIR LEPTON, The World's Sixth Sense^®^) which records the temperature of the tissue throughout the scan. An optimal Focal Plane Array (FPA) is maintained at 35°C to minimize the environmental factors and improve the image quality. In addition, to compensate for and minimize the intra and inter-patient thermogram variations and other factors, the device performs Flat Field Correction (FFC) before each scan, improving the pixel sensitivity and achieving a uniform response across the entire image.

The size of the field of view is 6.5 ^*^ 4 cm.

### The scan protocol

As mentioned previously, before each scan, the thermal camera sensor performs a self-calibration to ensure a reliable and accurate thermal reading. As the HT Vista system measures the difference in the temperatures between the mass and the adjacent healthy skin, and the probe is held on the skin to create a controlled microenvironment, factors such as ambient temperature and humidity are less significant. In addition, the clipping of the scan area does not affect the scan as both areas of interest are scanned, and the clinician does not need to wait after clipping before starting the scan.

Heat transfer rate acquisition was recorded by placing the probe in direct contact with the examined site (including the lesion and adjacent healthy skin) for 50 s with 10 s of heating and 40 s of cooling. During this time, temperature measurements were recorded by the thermal camera. Next, the clinician marked two sites on the optical image of the scanned area displayed on the touch screen. The data obtained was then uploaded to the HT Bioimaging cloud and analyzed using the HT Vista machine learning algorithm. During the validation study, the device provided the results immediately.

### Study population

The study population included dogs that presented to enrolling veterinary hospitals with a dermal or subcutaneous mass. Verbal informed consent was obtained from all owners. In addition, the owners were present during the procedure. The results of the scan did not affect subsequent diagnostics or treatments.

The inclusion criteria included an external subcutaneous or cutaneous mass that had adjacent healthy tissue that could be measured and imaged by the device, and it was considered safe for the dog to undergo a fine needle aspirate.

Exclusion criteria included certain tumor types and locations. Mammary tumors were excluded, as they are challenging to diagnose on cytology (24). Testicular tumors were excluded because of the absence of healthy tissue adjacent to the lesion. Facial tumors were excluded over the concern that the light source would be too close to the eyes and that the dogs would require sedation in order to heat and image the tumor. Severely ulcerated tumors were also excluded. If the dog moved during the scan or there was a technical problem, a second scan was performed. Only the results from the second scan were included in cases when two scans were performed.

### Data collection

Dogs were restrained (restraining was the only immobilization method used), and the lesion site and adjacent healthy tissue were clipped free of hair and scanned by the device. After the scan, the tested mass was aspirated and sent to an external clinical pathologist, who was blinded to the results obtained by the device. In some cases, surgery was subsequently performed, and a histopathologic diagnosis was obtained. In cases where the histopathologic diagnosis differed from the cytologic diagnosis, the pathology-derived diagnosis was considered the definitive diagnosis.

All dogs were monitored for adverse effects such as local irritation or edema.

Demographic information, tumor measurements, and lesion location were analyzed using commercially available software (Microsoft Excel).

### Study design

#### Feature generation and selection

In the feature generation phase, tens of features were generated based on the physical properties of the tissue and the mathematical properties of the signals. The feature generation phase also included a carefully chosen mechanism for combining the mass and the healthy tissue data. In the feature selection phase, we used statistical tools as well as optimization techniques to identify three leading features for the algorithm. The model selection phase included many standard machine learning models and cross-validation tests. The model that was eventually chosen was based on a linear combination of the three selected features, describing the thermal decay of the scan. All features were normalized to eliminate possible variances between patients and anatomical areas.

#### Training

Supervised machine learning models were applied in the training phase. We used optimization techniques to find the best possible parameters of the chosen model. The data for the training cohort, including the diagnosis and the thermal signals produced during the scan, were used to train the HT Vista machine learning algorithm. Additionally, we engaged in widely used learning approaches to increase the robustness of the training (i.e., leave-one-out and cross-validation).

#### Validation

After classifying the lesions by the device, the performance of the fixed algorithm was assessed. The prediction results were compared to the diagnosis obtained from cytology or histopathology, as explained above.

### Statistics

The overall accuracy, sensitivity, specificity, positive predictive value, and negative predictive value of the model in classifying tumors as benign or malignant were assessed. The confusion matrix was calculated for the training set using a Leave-One-Out mechanism. Then, a fixed model was employed to evaluate its performance in the validation set. Confusion matrix parameters (overall accuracy, sensitivity, specificity, positive predictive value, and negative predictive value) were calculated and compared to the train set. Statistical analysis was performed using the sci-kit-learn 1.1.0 package in Python 3.9. (25).

## Results

### Training cohort results

The study included 147 dogs with 233 masses. Sixty-six were mixed-breed dogs and 81 were purebred dogs. Purebred dogs included Labrador retrievers (nine dogs), pit bull terriers (eight dogs), and golden retrievers (six dogs). All other breeds had less than four dogs each. There were 39 intact female dogs, 38 spayed female dogs, 43 intact male dogs, and 27 castrated male dogs. The median age was 10 years, ranging between 1 and 16 years.

Of the 147 dogs, 97 dogs had one lesion sampled, 56 dogs had two lesions sampled, 42 dogs had three lesions sampled, 20 dogs had four lesions sampled, and three dogs had six lesions sampled. The diagnosis was based on cytology in 174 cases. Histopathology was performed in 59 cases. Six cases had both cytology and histopathology performed, resulting in the same diagnosis.

The training cohort included a wide range of both benign and malignant tumors. Forty-seven masses were classified as malignant lesions based on their cytology or histopathological diagnosis, and 186 were diagnosed as benign (Table 1).

**Table 1:** Results of the cytology and histology diagnosis of the lesions included in the training study.

**Tumor type**				
Benign		**Cytology**	**Histopathology**	**Total**
	Benign epithelial/adnexal cyst/tumor	33	9	42
	Benign melanoma		3	3
	Fibroma		2	2
	Hemangioma		5	5
	Histiocytoma		3	3
	Inflammatory process	6	3	9
	Lipoma	103	7	110
	Scar tissue		1	1
	Sebaceous adenoma	6	5	11
**Benign total**		**148**	**38**	**186**
Malignant	Malignant melanoma	2		2
	Mast cell tumors	11	14	25
	Plasma cell tumor	1		1
	Carcinoma	1	2	3
	Soft tissue sarcoma	11	3	14
	Undifferentiated neoplasia		2	2
**Malignant total**		**26**	**21**	**47**

The results were compared to the pathology reports. In total, 183 lesions were correctly classified, 37 as malignant and 146 as benign, while 50 were misclassified. Forty were classified as false-positive and 10 as false-negative. The overall accuracy, sensitivity, specificity, positive predictive value, and negative predictive value of the training cohort were 79%, 79%, 78%, 48%, and 94%, respectively. These numbers were computed using a Leave-One-Out mechanism. This differs from the validation phase, in which the algorithm was fixed and compared to the pathology results.

### Validation cohort results

This validation cohort included 299 dogs with 525 masses. One hundred and sixty-nine were mixed-breed dogs, and 130 were purebred dogs. Purebred dogs included Labrador retrievers (12 dogs), boxers (nine dogs), golden retrievers (eight dogs), shih tzus (seven dogs), pit bull terriers (six dogs), German shepherd dogs (five dogs), and four dogs from each of the following breeds: Australian shepherd, beagle, border collies, poodle, and Yorkshire terrier. All other breeds had less than four dogs each. There were 24 intact female dogs, 140 spayed female dogs, 28 intact male dogs, and 107 castrated male dogs. The median age was 9 years, ranging between one and 15 years.

**Figure 1:**
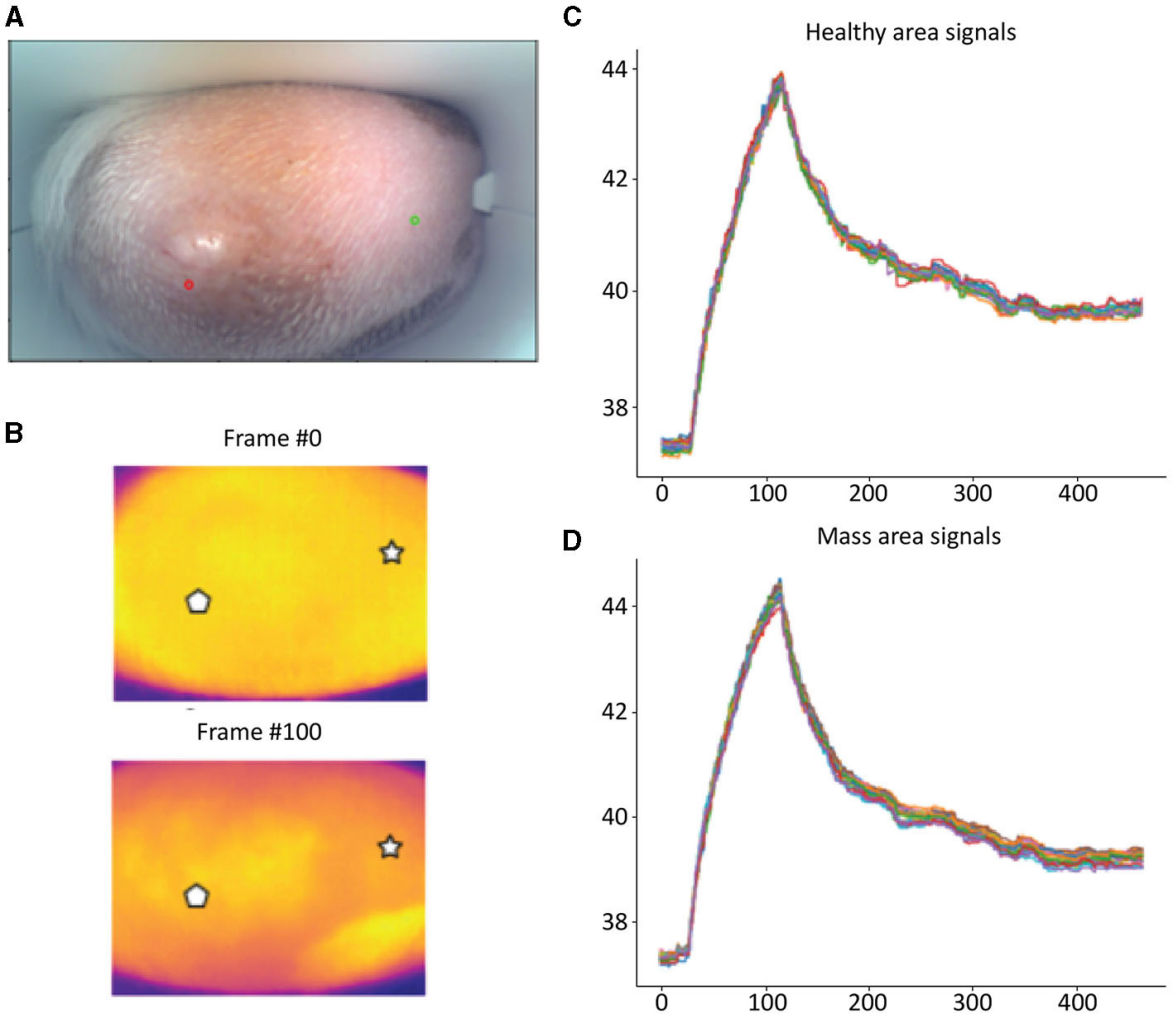
Mast cell tumor; **(A)** Optic image of a mast cell tumor from the scanner (Healthy site–green circle, mass site–red circle). **(B)** Thermal images from two time points (Frame 0 and Frame 100). **(C)** Measured temperatures from the healthy marked site over time, including basal, heating, and cooling temperature. **(D)** Measured temperatures from the mass marked site over time, including basal, heating, and cooling temperature. The thermal images do not show a difference between the healthy and malignant tissue. The measured temperatures in the graph show that the malignant tissue cooled down much faster than the healthy tissue.

**Figure 2:**
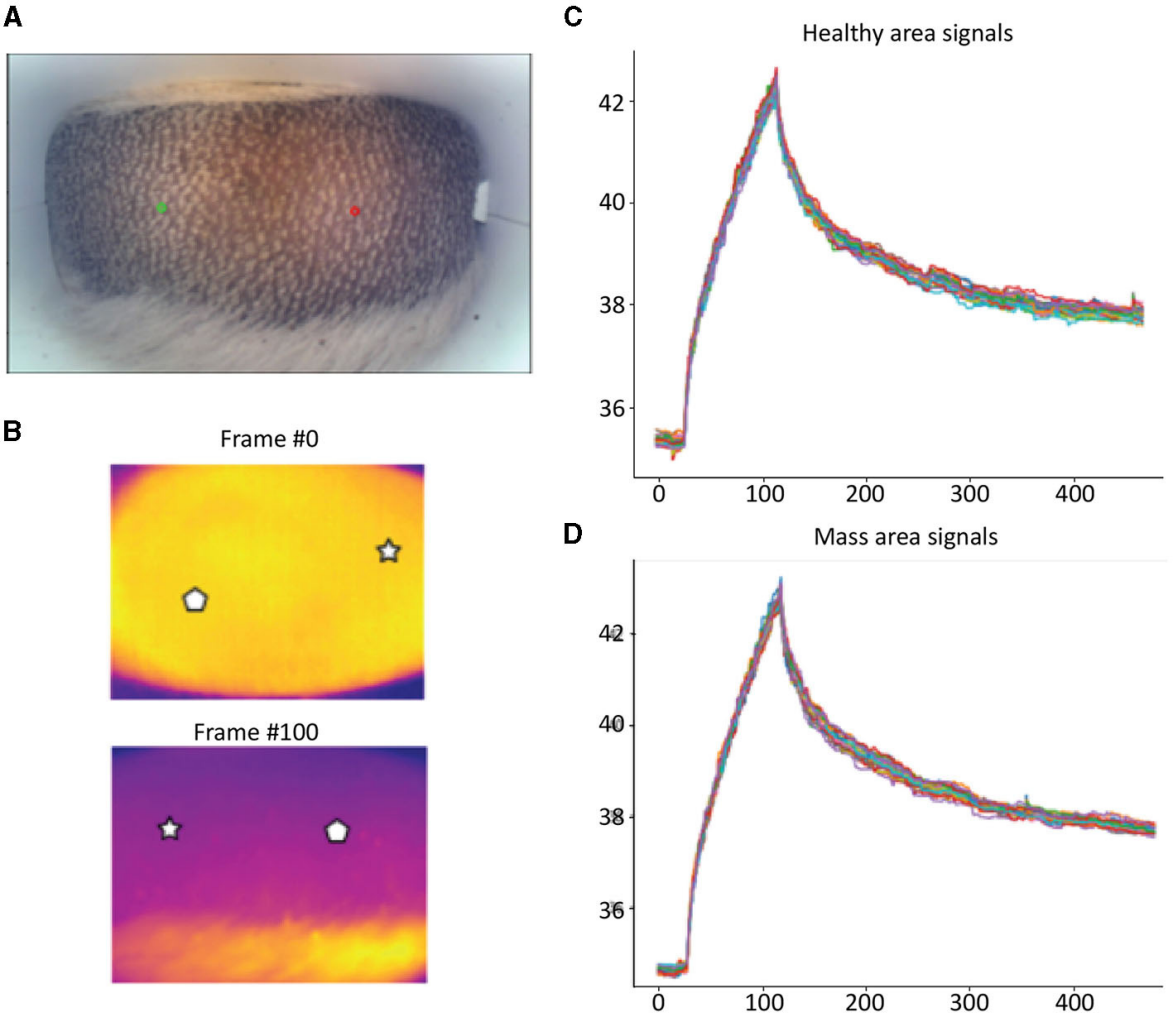
Lipoma; **(A)** Optic image of lipoma from the scanner (Healthy site—green circle, mass site—red circle). **(B)** Thermal images from to time points (Frame 0 and Frame 100). **(C)** Measured temperatures from the healthy marked site over time, including basal, heating, and cooling temperature. **(D)** Measured temperatures from the mass marked site over time, including basal, heating, and cooling temperature. The thermal images do not show a difference between the healthy and malignant tissue. The measured temperatures in the graph show that the lipoma did not heat up as much but cooled down in a similar fashion to the healthy tissue.

**Figure 3:**
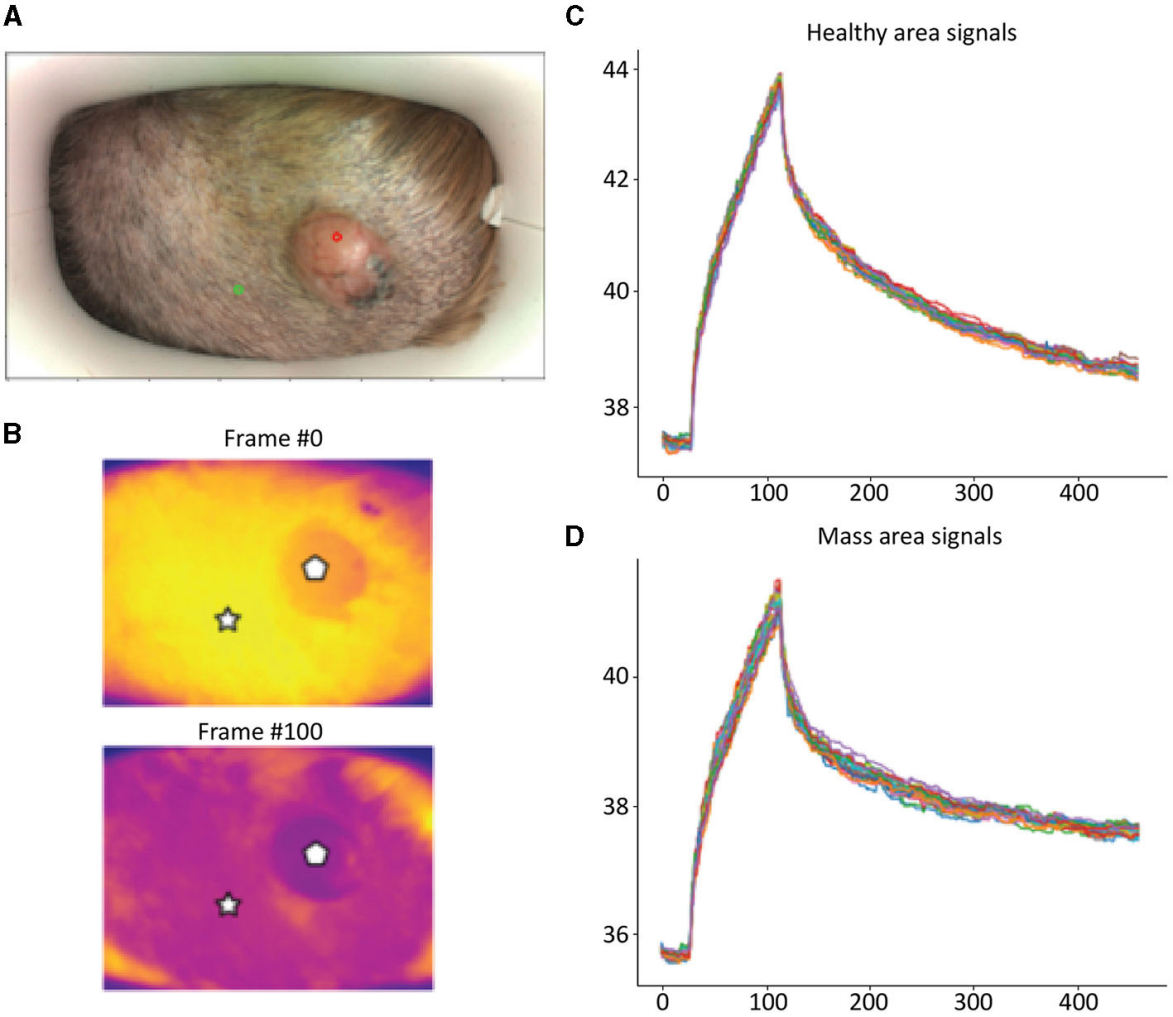
Basal Cell Tumor; **(A)** Optic image of basal cell tumor from the scanner (healthy site—green circle, mass site—red circle). **(B)** Thermal images from to time points (Frame 0 and Frame 100). **(C)** Measured temperatures from the healthy marked site over time, including basal, heating, and cooling temperature. **(D)** Measured temperatures from the mass marked site over time, including basal, heating, and cooling temperature. The thermal images show a difference between the healthy and benign tissue. The tumor is red and did not heat up as much as the healthy tissue. The measured temperatures in the graph show that the basal cell tumor and the healthy tissue elevated to different temperatures and did not heat up in a similar manner. The thermal images show a difference between the healthy and benign tissue. The tumor is red and did not heat up as much as the healthy tissue. The measured temperatures in the graph show that the basal cell tumor and the healthy tissue elevated to different temperatures and did not heat up in a similar manner.

Detailed examples of the data obtained during the scan in a mast cell tumor, lipoma, and basal cell tumor are shown in Figures 1–3, respectively. The data acquired includes optical and thermal images of the mass and the adjacent healthy tissues, alongside the temperature measurements at baseline and throughout the heating process.

Of the 299 dogs, 181 dogs had one lesion sampled, 61 dogs had two lesions sampled, 23 dogs had three lesions sampled, 21 dogs had four lesions sampled, nine dogs had five lesions sampled, and four dogs had six lesions sampled, resulting in a total of 525 scanned lesions. Cytology was non-diagnostic in 94 masses, resulting in 431 masses from 248 dogs with diagnostic samples. The study included a wide range of both benign and malignant tumors. Fifty-three masses were classified as malignant lesions based on their cytology or histopathological diagnosis, and 378 were diagnosed as benign lesions. Additional histopathology was performed on 41 of the lesions. One case remained non-diagnostic after histopathology (Table 2).

**Table 2:** Results of the cytology and histology diagnosis of the lesions included in the validation study.

**Tumor type**	**Tumor class**			
Benign		**Cytology**	**Histopathology**	**Total**
	Benign epithelial/adnexal cyst/tumor	85	9	94
	Benign melanoma	2	2	4
	Calcinosis circumscripta	1		1
	Histiocytoma	2	1	3
	Hyperplasia	3		3
	Inflammatory process	20	2	22
	Lipoma	250		250
	Perineal adenoma	1		1
Benign total		364	14	378
Malignant	Carcinoma	2		2
	Mast cell tumor	19	19	38
	Soft tissue sarcoma	5	8	13
Malignant total		26	27	53

Using the fixed machine learning classifier, each scanned site was classified as either benign or as a mass that requires further investigation. The results were compared to the pathology reports. In total, 298 lesions were correctly classified, 45 as malignant and 253 as benign, while 133 were misclassified. One hundred and twenty-five were classified as false-positive and eight as false-negative. The overall accuracy, sensitivity, specificity, positive predictive value, and negative predictive value of the device and algorithm in this study were 69%, 85%, 67%, 26%, and 97%, respectively.

Table 3 displays the diagnosis list with the predicted results from the HT Vista device. The eight false negative lesions were all diagnosed as mast cell tumors. Three of those tumors were surgically removed and diagnosed on histopathology with low-grade tumors. Thirty-eight mast cell tumors were scanned in the study, and 18 of them had pathology performed. Of the mast cell tumors with histopathology, 13 were low-grade and five were subcutaneous tumors. All of the carcinomas and sarcomas were classified correctly by the device. Histopathology was performed in nine soft tissue sarcomas; seven were grade I, one was a grade II, and one was a grade III tumor.

**Table 3:** Results of the cytology and histology diagnosis and the HT real-time predication of the lesions included in the study.

		**HT real-time prediction**		
**Benign/malignant**	**Tumor type**	**Benign**	**Malignant**	
Benign	Benign epithelial/adnexal cyst/tumor	37	57	94
	Benign melanoma	3	1	4
	Calcinosis circumscripta	0	1	1
	Histiocytoma	1	2	3
	Hyperplasia	1	2	3
	Inflammatory process	5	17	22
	Lipoma	206	44	250
	Perineal adenoma	0	1	1
Benign total		**253**	**125**	**378**
Malignant	Carcinoma	0	2	2
	Mast cell tumor	8	30	38
	Soft tissue sarcoma	0	13	13
Malignant total		8	45	53
Grand total		**261**	**170**	**431**

One hundred twenty-five lesions were classified as false positives. These lesions included 57 benign epithelial tumors and adnexal cysts; 44 lipomas; 17 inflammatory lesions; two cases each of hyperplasia and histiocytoma; and one case each of benign melanoma, a calcinosis circumscripta, and a perianal adenoma.

Three cases had an adverse effect. All were cases of mast cell tumors that were aspirated following the scans. In two cases, the adverse effects observed included erythema and swelling at the mass site that resolved with treatment with systemic diphenhydramine and prednisone. The third case vomited twice in the evening following scanning and aspiration and was treated with diphenhydramine and famotidine. All of these adverse events were resolved without lasting consequences.

## Discussion

AI-driven medical devices are becoming more common in veterinary medicine (26–28). They are used to solve problems of high logical or algorithmic complexity, ranging from diagnosis and disease detection to making reliable predictions and reducing medical errors (29). Screening tests in veterinary medicine are performed to detect potential health disorders or diseases with a goal of early detection, to reduce the risk of disease, or detect it early enough to treat it most effectively (30). This study has shown that this novel system could be used as a screening tool and decision support tool for the everyday diagnosis of cutaneous and subcutaneous masses in general practice, enabling clinicians to differentiate between benign lesions and those requiring additional diagnostics.

The algorithm training process used data from dogs with masses diagnosed as either benign or malignant and resulted in a fixed algorithm used in the validation portion of the study. As the goal of an optimal screening test is a high negative predictive value and high sensitivity, the algorithm was designed accordingly.

The validation portion of the study differed in that all dogs that were presented for scanning and diagnosis were included in the study, and the scan results were available immediately. Therefore, some masses [94 (18%)] were excluded because the cytology was inconclusive. This is consistent with previous reports and supports the need for real-time determination of the need to further diagnose and assess masses (9).

The accuracy of this system was 69%, correctly classifying 298 out of 431 masses. Forty-five of the fifty-three malignant tumors were classified as true positives, including carcinomas, sarcomas, and mast cell tumors. All of the carcinomas and sarcomas and 30 of the 38 mast cell tumors were classified correctly. The eight false negative cases were all mast cell tumors. Three of these eight tumors had histopathology performed; all were low-grade tumors. Although mast cell tumors are considered malignant tumors, the diversity of these tumors, including different grades, might also cause differences in their thermal conductivity, causing the less aggressive tumors to act as benign lesions (8). Additional evidence was reported by the Oncology-Pathology Working Group in the Summary and Subgroup Recommendations for Grading of Canine Cutaneous Mast Cell Tumors, revealing that low-grade/grade I mast cell tumors have an excellent prognosis with virtually no tumor-related deaths reported in these cases (31).

Two hundred and fifty-three cases were classified as true negatives. The system's algorithm was programmed to give a high degree of certainty when the mass is declared benign, reflected by the high negative predictive value. This comes at the expense of relatively low specificity, resulting in an increased number of false positives. One hundred twenty-five cases were classified as false positives, mainly including benign epithelial tumors, adnexal cysts, lipomas, and inflammatory lesions. There are several explanations for these results. Deep tumors over 0.5 cm deep may not heat sufficiently and therefore require a change of the heat source configuration (e.g., the wavelength and penetration characteristics). The cellular debris within epidermal and adnexal cysts may heat differently than solid tissue and may lead to false positive results in these cases. Furthermore, in cases of primary and secondary inflammation, as in epidermal and adnexal cysts (8), the inflammatory component may react to the thermal excitation in an abnormal manner, similar to malignant tissues, thus resulting in a false positive classification. All positive scans (true positive and false positive) warrant additional diagnostics. As this device is designed as a screening tool, false positive results are expected. Although some of the false positives will be benign tumors, others will benefit from additional diagnostics, as even lesions that are not malignant may require treatment (infection and inflammation). The number of false positives is preferable to a higher number of false negatives, which would cause clinicians to send home animals with malignant tumors. Therefore, this system enables the clinician to justify continuing to diagnose these suspicious masses with either an aspiration and/or a biopsy and not take the wait-and-see approach.

Several adverse effects were reported after scanning and performing fine needle aspirates of mast cell tumors. The erythema and swelling of the mast cell tumors were most likely due to degranulation of the mast cells, i.e., Darier's sign (2). We assume that these adverse effects were most likely due to the aspiration event as this is a known adverse event with this procedure and not the scans, but this was not tested independently. The vomiting seen in one dog on the day of the aspirate may have been due to the procedure or other causes unrelated to the mast cell tumor.

The limitations of the study include the low number of malignant cases, as the study was performed almost entirely at local veterinary clinics and not specialty clinics, reflecting the cases that are seen there regularly. The system is designed to be a diagnostic screening tool in general practice; therefore, the clinics included in the study represent the target population. Another limitation is that we are comparing the adjacent healthy tissue to the tumor. As a result of the size of the scanner, it is possible that an area marked as healthy could be already compromised with malignant cells. The third limitation is that we had a combination of cytology and histology reports. As histology is the gold standard, it would be ideal if all of the masses were diagnosed based on histology. However, most owners would not cooperate if a biopsy was required for every mass that was included in the study.

Future directions include additional uses for the device, staging for malignant tumors, and evaluating specific tumor types for their characteristics. Artificial intelligence algorithms are data-driven; therefore, the performance should improve as more data is collected.

In conclusion, we validated this novel screening and decision support tool for the clinical management of cutaneous and subcutaneous masses in dogs, using dynamic heat diffusivity and analysis of the produced signal utilizing advanced machine learning.

## Data availability statement

The original contributions presented in the study are included in the article/supplementary material, further inquiries can be directed to the corresponding author.

## Ethics statement

The animal studies were approved by the Israeli Health Ministry Ethical Review Board Committee (PET 2023-03-NE). The studies were conducted in accordance with the local legislation and institutional requirements. Written informed consent was obtained from the owners for the participation of their animals in this study.

## Author contributions

All authors contributed with planning of the study, case accrual, work on the algorithm, and manuscript preparation.
